# Redox-Driven Formation of Mn(III) in Ice

**DOI:** 10.1021/acs.est.4c03850

**Published:** 2024-08-17

**Authors:** Tao Chen, Tra My Bui Thi, Tao Luo, Wei Cheng, Khalil Hanna, Jean-François Boily

**Affiliations:** † École Nationale Supérieure de Chimie de Rennes, CNRS, ISCR-UMR 6226, Université de Rennes, F-35000 Rennes, France; ‡ College of Resources and Environmental Science, South-Central University for Nationalities, Wuhan 430074, P. R. China; § Department of Chemistry, Umeå University, SE-901 87 Umeå, Sweden

**Keywords:** ice, manganese, biogeochemical cycle, redox

## Abstract

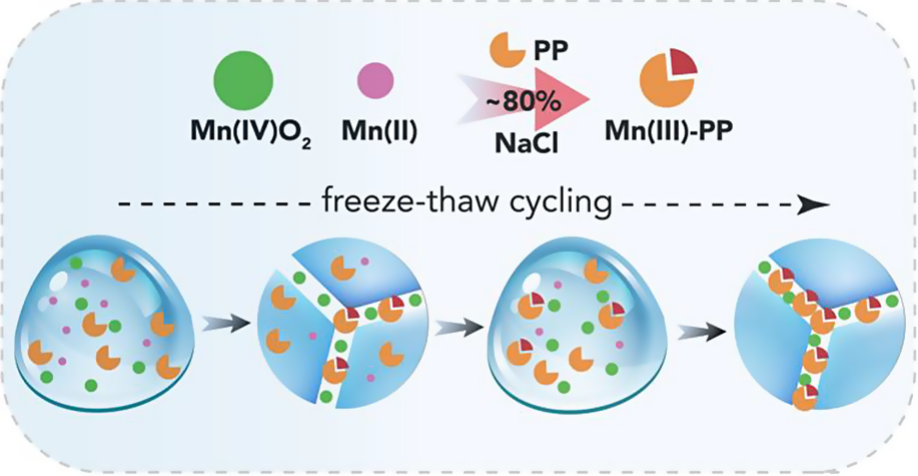

Redox-driven reactions involving Mn(II) species adsorbed at Mn(IV) oxide surfaces can release Mn(III) in the form of dissolved Mn(III)–ligand species in natural waters. Using pyrophosphate (PP) as a model ligand, we show that freezing accelerates and enhances Mn(III) formation in the form of Mn(III)–PP complexes. This freeze-promoted reaction is explained by the concentration of Mn(IV) oxides and solutes (Mn(II), Na^+^, and Cl^–^) into the minute fractions of liquid water locked between ice (micro)crystals - the Liquid Intergrain Boundary (LIB). Time-resolved freezing experiments at −20 °C showed that Mn(III) yields were greatest at low salt (NaCl) content. In contrast, high salt content promoted Mn(III) formation through chloride complexation, although yields became lower as the cryosalt mineral hydrohalite (NaCl·2H_2_O) dehydrated the LIB by drawing water into its structure. Consecutive freeze–thaw cycles also showed that dissolved Mn(III) concentrations increased within the very first few minutes of each freezing event. Because each thaw event released unreacted PP previously locked in ice, each sequential freeze–thaw cycle increased Mn(III) yields, until ∼80% of the Mn was converted to Mn(III). This was achieved after only seven cycles. Finally, temperature-resolved freezing experiments down to −50 °C showed that the LIB produced the greatest quantities of Mn(III) at −10 °C, where the volumes were greater. Reactivity was however sustained in ice formed below the eutectic (−21.3 °C), down to −50 °C. We suspect that this sustained reactivity was driven by persistent forms of supercooled water, such as Mn(IV) oxide-bound thin water films. By demonstrating the freeze-driven production of Mn(III) by comproportionation of dissolved Mn(II) and Mn(IV) oxide, this study highlights the potentially important roles these reactions could play in the production of pools of Mn(III) in natural water and sediments of mid- and high-latitudes environments exposed to freeze–thaw episodes.

## Abstract 2

Freezing drives the production of Mn(III) by promoting interactions between dissolved Mn(II) and Mn(IV)-oxide surfaces.

## 1 Introduction

Manganese (Mn) is the third most abundant-redox active^1^ metal in Earth’s crust,^2^ and is of great importance to many redox-driven biogeochemical processes.^3^ In natural aquatic systems, Mn(II), Mn(III), and Mn(IV) species are present over a wide range of environmental redox conditions, and undergo rapid cycling between these states.^4^ Mn(IV) forms strongly oxidizing minerals (e.g., cryptomelane (α-MnO_2_), pyrolusite (β-MnO_2_), and birnessite (δ-MnO_2_)),^5^ while Mn(II) is primarily in the form of dissolved aqueous species.^6,7^ Mn can also form minerals, such as rhodochrosite (MnCO_3_), in reducing environments.^8^

Interactions between Mn(II) species and Mn(IV) oxides have been the focus of many studies following Mn(II)-induced heavy metal sorption^9−12^ and phase transformation^13−15^ reactions. Mn(III) species have, on the other hand, not received as much attention given their strong propensity to disproportionate into Mn(IV) oxides and Mn(II) species, and for precipitating into oxyhydroxides (e.g., feitknechtite (β-Mn(III)OOH), Manganite (γ-Mn(III)OOH)) or mixed valent Mn(III, IV) oxides (e.g., hausmannite (Mn_3_O_4_)).^13−16^ Still, Mn(III) can form stable aqueous species under acidic conditions and, under circumneutral conditions, as (in)organically complexed species.^17−20^

In nature, soluble Mn(III) can be released by the abiotic and biotic oxidation of Mn(II)^3,21−24^ or by the reduction of MnO_2_.^25,26^ This can take place through a one electron transfer reaction,^27^ as well as through ligand-promoted nonreductive^28,29^ and reductive dissolution^5,30−32^ of Mn-bearing minerals. The potentially important roles that Mn(III) species play in nature can be attributed to their prolonged lifetime when bound to (in)organic ligands (L). These Mn(III)–L species can, as a result, secure Mn(III) transport across redox gradients in nature.^7,33,34^ For instance, Mn(III)–L complexes represent the majority of the total dissolved Mn(III) pool in anoxic, suboxic and oxic environments of oceanic and estuarian sediments, where they can be present up to tens of micromolar level.^19,35,36^ Mn(III)–L species also account for the great majority of dissolved Mn species in suboxic waters of the Black^19^ and Baltic^37^ seas, and 90% of soluble Mn in sediment porewaters of the Laurentian Trough.^33^ Soluble Mn(III) was even detected at every stage in a water treatment work in England, particularly when Mn oxide solids interacted with natural organic matter in the clarifier sludge.^38^

Although the composition of complexing ligands is still not well-known, compounds responsible for stabilizing Mn(III) include naturally occurring carboxylate- or phosphonate-bearing moieties in natural ligands.^17^ These ligands include organic chelates, for instance natural organic matter (e.g., humic substances),^39^ ethylenediaminetetraacetate (EDTA),^40^ citrate,^30^ and siderophores (e.g., desferrioxamine B),^41^ as well as polyphosphates.^17,29,42,43^ These ligands, especially polyphosphates, are likely important contributors to Mn cycling in nature.^33^ The reactive Mn(III), serving as both electron donor and acceptor, may even possibly be linked to the cycling of carbon, arsenic, iron, and sulfur.^44,45^

The natural occurrence of soluble Mn(III) could, however, be altered in frozen environments of the Cryosphere. This chemistry is driven by minute fractions of liquid water locked between ice microcrystals in polycrystalline ice formed above the eutectic temperature of NaCl-bearing water (−21.3 °C). Particulate matter and solutes concentrated into this Liquid Intergrain Boundary (LIB) can react at different rates and generate different products than in liquid water.^46−51^ This could, as such, be the case for the important comproportionation reactions that control Mn redox geochemistry. Evaluating the role that freezing could play on geochemical reactions is particularly relevant noting that about two-thirds of Earth’s entire freshwater supply is stored in ice and glaciers, and that a great portion of the water in mid- to high-latitude regions can freeze or even undergo several freeze–thaw episodes during cold seasons.^52^

In this study, we addressed the roles that freezing plays in the formation of Mn(III) by reacting dissolved Mn(II) and birnessite (MnO_2_), an important nanolayered Mn(IV)-oxide nanomineral. Both commonly occur in water columns and sediments at concentrations of tens of microns.^19,53^ We used pyrophosphate (PP) as redox-inert chelating ligand to stabilize Mn(III) in the form of aqueous Mn(III)–pyrophosphate (Mn(III)–PP) complexes.^42^ The focus on PP was also motivated by its presence in aquatic environments, such as in marine particulate and estuary sediments and even in industrial discharge, present in the order of tens to hundreds of micromoles.^36,54−56^ By exploring how temperature, ionic strength, pH, and freeze–thaw cycling affected Mn comproportionation reactions in the LIB, we showed that freezing greatly enhances reaction rates and yields of Mn(III) species. These findings should contribute to understanding Mn cycling as species migrate through interconnected bodies of water in the cryosphere and the hydrosphere, including those from fresh to oceanic waters.

## 2 Experimental Methods

### 2.1 Solutions and Materials

Stock solutions of manganese(II) chloride tetrahydrate, and sodium pyrophosphate decahydrate salts (Sigma-Aldrich) were prepared in ultrapure water. Manganese(III) acetate dihydrate (Sigma-Aldrich) was used as received without purification. Standard solutions of premade 1 M NaOH or 1 M HCl (Sigma-Aldrich) were used to adjust the pH of the solutions. Acid birnessite was synthesized by reacting KMnO_4_ and HCl in boiling water.^57^ The resulting suspension was purged from dissolved atmospheric CO_2_(g) using a stream of N_2_(g), then stored in the dark at 4 °C. The study of Li et al.^57^ contains a detailed report on the synthesis and purification procedures, as well as salient physicochemical properties of the high specific surface area (85 m^2^/g) MnO_2_ nanoparticles used for this work.

### 2.2 Batch Experiments

Batch experiments were conducted in transparent polyethylene centrifuge tubes containing 10 mL of pH ∼ 7 solutions of 250 μM pyrophosphate and 50 μM MnCl_2_ solutions in 0–50 mM NaCl. The theoretical PP:Mn(III) = 2.5:1 ratio, assuming full conversion of Mn, chosen for this work ensured that all Mn(III) species can in most cases be complexed with PP at circumneutral pH,^42^ as confirmed by thermodynamic calculations of the aqueous solutions using the program PHREEQC (Figure S4, Table S2).^58^

Reactions were initiated by adding a 50 μM MnO_2_(s) to the solution. The resulting suspensions (4.35 mg/L MnO_2_(s)) were immediately shaken manually, each sealed with a septum. Based on control experiments in the dark at 25 °C, the low MnO_2_ suspension densities used in this work were not sufficiently large to initially alter suspension pH. Samples were thereafter frozen to −10, −20, −30, or −50 ± 0.1 °C by submerging the test tubes into precooled liquid ethanol in a circulation bath. Samples were completely frozen within ∼2 min. From the uniform distribution of color along the tubes of the frozen samples, we infer that MnO_2_ particles were well-dispersed throughout the frozen suspensions. Frozen samples were then periodically withdrawn from the ethanol bath and thawed in lukewarm water (30–40 °C) for ∼5 min. This thawing period was not sufficiently long to react Mn(III) species any further, as we could assess by comparing reaction yields in liquid water. The resulting aqueous suspensions were then filtered (0.2 μm filter) and the supernatant analyzed for Mn(III).

Freeze–thaw (F–T) cycling experiments were performed in the same fashion as the freezing experiments, except that they consisted of 45 min cycles, each comprising a period of 20 min in liquid water (25 °C), followed by another 20 min period in ice (−20 °C), and a final ∼5 min thawing period in lukewarm water. Two sets of freeze–thaw cycling experiments were performed. One set of experiments in 0.5 mM NaCl was carried out for 7 consecutive cycles. Another set of experiments at 12 different ionic strengths within the 0.5–500 mM NaCl range, were analyzed for Mn(III) after 1, 3, and 7 F–T cycles.

All batch freezing experiments were performed in the dark without controlling the atmosphere of the headspace, unless otherwise noted. Contributions from oxygen-driven formation of Mn(III) were negligible, as additional sets of experiments showed no difference in Mn(III) content in samples reacted under aerobic and anaerobic conditions (Figure S1). All experiments were repeated at least twice, and with a reproducibility of ∼5% in Mn(III) concentrations.

### 2.3 Mn(III) Concentration Determination

Concentrations of Mn(III)–PP complexes were measured by UV–vis spectrophotometry. From the aqueous speciation of mixed Mn(III)/PP solutions (Figure S4, Table S2), Mn(III) should be predominantly in the form of the MnPP_2_^5–^ complex. A 10 mM Mn(III) and 40 mM PP stock solution used to prepare standards was made by adding a weighed aliquot of dry manganese(III) acetate dihydrate powder to a 40 mM sodium pyrophosphate decahydrate solution at pH ∼ 8.^29,40,42^ The solution was equilibrated with magnetic stirrer, and the pH shifted to ∼7. The resulting standard solution was then stored in the dark in a refrigerator at 4 °C to mitigate PP decomposition. The experimental work was completed well within any significant decomposition of the standard Mn(III)–PP solutions, which have a half-life of several hundreds of days.^40,42^

The UV–vis spectra of all Mn(III)–PP standards and analytes were collected in the 200–800 nm range in a 10 mm quartz cell using an ultraviolet–visible (UV–vis) spectrophotometer (Cary-50, Varian). All absorbances were offset to 0 in the 600–800 nm range to correct minor shifts in absorbances. Mn(III) concentrations were then determined using absorbance values at 258 nm (linear fit of calibration curve with *r*^2^ > 0.99). Note that absorbances were not affected by pH, given a previous report on the molar absorbances for Mn(III)–PP complexes.^40^

### 2.4 Mn(II) Adsorption on MnO_2_

Mn(II) adsorption experiments were conducted in 10 mL test tubes containing 50 μM MnCl_2_ and 50 μM MnO_2_(s) (4.35 mg/L) in the presence and absence of 250 μM PP in 0.5 mM NaCl at pH 7. Experiments were carried out at 25 and −20 °C for 1 h. Those at −20 °C were followed by a ∼5 min period of thawing in lukewarm water. All resulting aqueous suspensions were thereafter filtered (0.2 μm) and analyzed for dissolved Mn using inductively coupled plasma-atomic emission spectrometry (ICP-AES, 725-ES, Varian).

Changes in surface Mn oxidation state on reacted MnO_2_(s) were probed by X-ray photoelectron spectroscopy (XPS). Solids collected from batch adsorption experiments were first washed with N_2_(g)-sparged Milli-Q water (18.2 MΩ·cm) to remove residual dissolved Mn and pyrophosphate, then dried under a stream of N_2_(g). Spectra of the resulting dry solids were thereafter acquired using a Kratos Axis Ultra electron spectrometer equipped with monochromatic Al K_α_ X-ray (1486.7 eV) source operating at 10 mA and 15 kV. Mn oxidation states were evaluated by spectral fitting of the Mn 2p_3/2_ region using the approach of Ilton et al.^59^ Briefly, the procedure involves a combination of Gaussian/Lorentzian (G/L) peaks (Figure S6) obtained from solid-state references (Table S3) for Mn(II) (MnCl_2_), Mn(III) (MnOOH; Manganite), and Mn(IV) (MnO_2_; pyrolusite).^59,60^ All fitting procedures were performed on CasaXPS,^61^ using a Shirley background on all spectra.

### 2.5 Raman Spectroscopy

The cryosalt mineral hydrohalite (NaCl·2H_2_O) formed by freezing NaCl-bearing solutions was probed by Raman spectroscopy. Spectra were acquired with a Renishaw InVie Qontor Raman spectrometer using a 532 nm continuous wave laser with a 50× Long Working Distance Objective Lens. Measurements were performed on single ∼10 μL droplets of 0.5–500 mM NaCl solutions dropped on a temperature-controlling microscope stage (Linkam Scientific THMS600). Temperature was then decreased from 25 to −20 °C at a rate of 5 °C/min using liquid nitrogen as the cooling agent. The microscope was then focused on the surface of the frozen droplet, and an optical image was obtained to identify the spatial distribution of the LIBs. Spectra were thereafter collected in the O–H stretching region (2600–4000 cm^–1^), with a total of 100 scans with the objective refocused on selected portions of LIBs.

## 3 Results and Discussions

**Figure 1:**
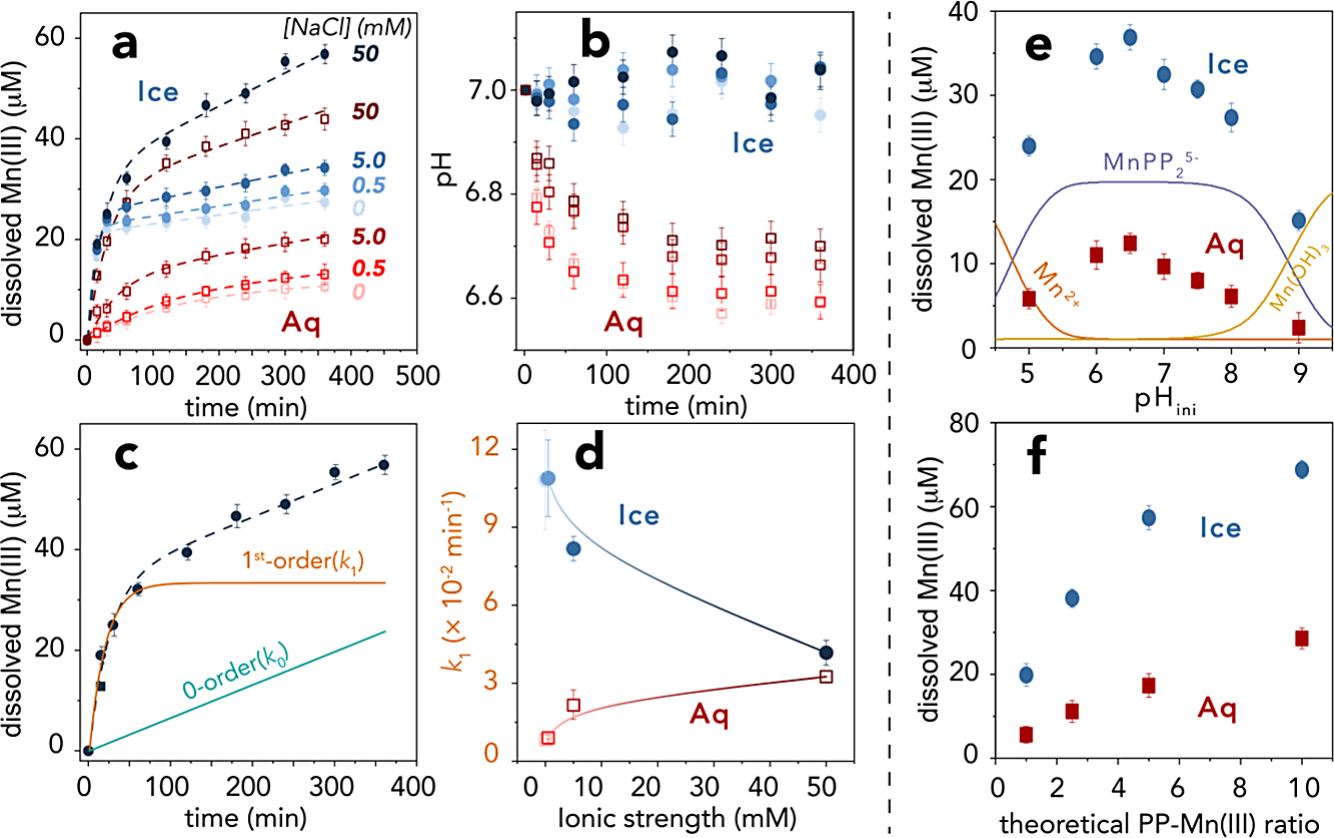
Mn(III) formation by comproportionation of 50 μM MnO_2_ (4.35 mg/L) and 50 μM Mn^2+^ in the presence of (a–d) 250 μM PP at pH_ini_ = 7 ± 0.05, and variations in Mn(III) concentrations versus (e) pH and (f) theoretical PP-Mn(III) ratio. All reactions in liquid water (Aq) were at 25 °C and in ice (Ice) at −20 °C. (a) Time-resolved Mn(III) concentrations (symbols) and kinetic models (dashed lines), (b) changes in pH in 0–50 mM NaCl, (c) detailed kinetic modeling in frozen 50 mM NaCl, and (d) first-order rate constants (symbols) with lines as a visual guide. (e) pH-dependent Mn(III) concentrations after 2 h of reaction in 0.5 mM NaCl against chemical speciation at equilibrium (Figure S4, Table S2). (f) PP:Mn(III) ratio dependence on Mn(III) after 2 h of reaction in 0.5 mM NaCl as a function of “theoretical PP-Mn(III) ratio” (i.e., full conversion of 50 μM Mn(IV) and 50 μM Mn(II) to 100 μM Mn(III)).

Time-resolved batch experiments (Figure 1a) showed that comproportionation reactions in ice (−20 °C) were ∼10 times faster and produced ∼2.5 times more Mn(III) than those in liquid water (25 °C) after 6 h of reaction. Also, from the unchanged values of pH ∼ 7, losses in proton concentration from the reactions (e.g., Mn^2+^ + MnO_2_(s) + 4H^+^ → 2 Mn^3+^ + 2H_2_O) were smaller than the buffering capacity of the system. In contrast, a shift in the pH from ∼6.8 to ∼6.6 in the unfrozen samples may have resulted from the progressive uptake of atmospheric CO_2_. This shift was, however, not sufficient to significantly alter aqueous speciation, as evaluated by thermodynamics. (Figures S3 and S4).

Mn(III) concentrations produced in liquid water scaled with ionic strength (Figure 1a). This can be well described using a zero- and first-order composite model (Figure 1c, Table S1):
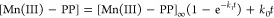
1where [Mn(III)–PP]_∞_ is the concentration of Mn(III)–PP at equilibrium, *k*_1_ is the first-order reaction rate constant, and *k*_0_ is the zero-order reaction rate constant. Contributions from the ligand-assisted dissolution of Mn(III) from MnO_2_ or abiotic oxidation of Mn^2+^ were ruled out (Figure S1). We ascribe (i) *k*_1_ to fast Mn^2+^ adsorption reactions with negatively charged MnO_2_ sites (point of zero charge, pH_pzc_ ∼ 2) in the stages of the reaction,^29,57^ and (ii) *k*_0_ to slower adsorption reaction on neutrally charged basal faces of MnO_2_ and potentially to diffusion to interlayer sites.^62^ Both rates scaled in liquid water with ionic strength (Figures 1d and S2), and this is supported by studies^63−65^ showing that Mn-chloride complexation facilitates electron transfer rates. In contrast, first-order Mn(III) formation rates (*k*_1_) in ice were ∼10 times larger than in liquid water at low ionic strength, yet nearly identical at 50 mM NaCl (Figure 1d). We attribute this enhancement at low ionic strengths to the locking of reactive species (MnO_2_, Mn^2+^ and PP) in the LIB after freezing. This locking caused first-order reactions to dominate in the first ∼40 min of freezing, initially producing ∼20 to 30 μM Mn(III) (Figure 1a).

**Figure 2:**
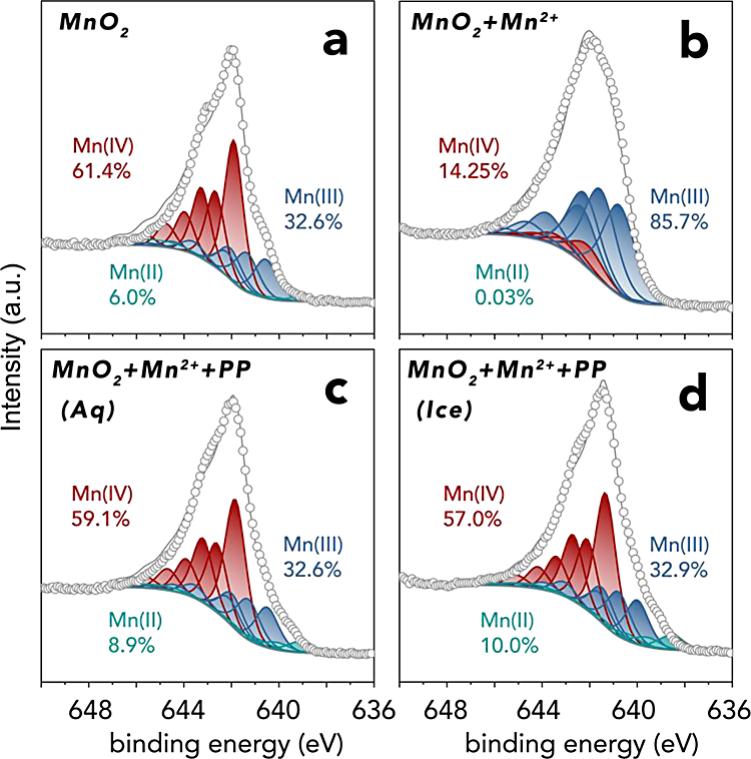
XPS spectra and fitting results of the Mn 2p_3/2_ region of (a) unreacted MnO_2_, (b) MnO_2_ reacted with Mn^2+^ in the absence of PP in liquid water at 25 °C, and (c-d) MnO_2_ reacted with Mn^2+^ and PP in (c) liquid water (Aq) at 25 °C and (d) in ice at −20 °C (Ice). Atomic % values of Mn oxidation states are shown for every sample.

To explain these results, we explored (i) Mn speciation using thermodynamic calculations (Figures S3 and S4, Table S2), as well as (ii) Mn(III) yields as a function of pH (Figure 1e) and Mn:PP ratios (Figure 1f). We make the following three key observations:

(i) From thermodynamics, we note that the main reactive species should be the free Mn^2+^ (42%) and the complexed MnPP^2–^ (58%) ions (Figure S3). The Mn^2+^ ion should consequently be the main adsorbing Mn species, given the low pH_pzc_ of the Mn(IV) oxide. From batch adsorption experiments (Figure S5), we find that these reactions began with an initial loading of ∼20 Mn/nm^2^. Mn(III) formed in both liquid water and ice via one-electron interfacial transfer of the adsorbed Mn^2+^ complexes to MnO_2_. This was confirmed by XPS (Figure 2), showing a substantial comproportionation of Mn(II) (6.0 → 0.0%) and Mn(IV) (61.4 → 14.3%) to Mn(III) (32.6 → 85.7%) in the absence of PP (Figures 2a,b), and that Mn(II):Mn(III):Mn(IV) remained relative unchanged during the ligand-assisted dissolution of the newly formed surface Mn(III) species by PP (Figure 2c,d). The following reactions:
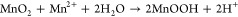
2
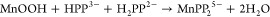
3explain speciation and oxidation changes undergone in the system.

(ii) Second, experiments showed that the largest concentrations of Mn(III) after 2 h were at pH 6–7 (Figure 1e). Smaller concentrations of Mn(III) produced at low and high pH can be explained by freeze-induced shifts in pH. More extreme pH converted MnPP_2_^5–^ to hydrolyzable PP species (Figure S4). We expect that these shifts occurred because excess H^+^ or OH^–^ ions in noncircumneutral solutions migrated to or from the LIB during freezing. These shifts could have also been accentuated by the differential partitioning of solute between ice and water,^66,67^ and even by the spontaneous disproportionation of Mn(III) below pH ∼ 5 (*E*_H_^0^_(Mn3+/Mn2+)_ = +1.56 V)^68^.

(iii) Third, we found that Mn(III) concentrations scaled with PP:Mn(III) ratios (Figure 1f). A PP:Mn(III) ratio of 2 was needed to convert Mn(III) to MnPP_2_^5–^ (Figure S4), and even higher ratio (PP:Mn(III) > 5) is usually required to fully stabilize Mn(III) at circumneutral pH.^42^ Previous work^29^ however showed that PP, just like other ligands,^69^ can promote Mn detachment from MnO_2_.

These results therefore support the concept that the freeze-driven concentration of PP into the LIB catalyzed Mn(III) production by both mitigating disproportionation^63^ and buffering pH. This was even the case at a PP:Mn(III) ratio of 1:1, where the freeze concentration effect increased PP:Mn(III) ratios to levels that generated ∼4 times more Mn(III) in ice than in liquid water. This Mn(III)-promoting freeze concentration effect became, however, less effective at larger PP dosages. One possible explanation for this nonlinear response to PP dosage could be that a fraction of unreacted PP species was trapped in ice. This explanation aligns with previous work^70^ showing that solids and selected solutes tend to be mostly rejected from ice crystals, while other ligands can also be readily incorporated into the ice lattice.^70^

**Figure 3:**
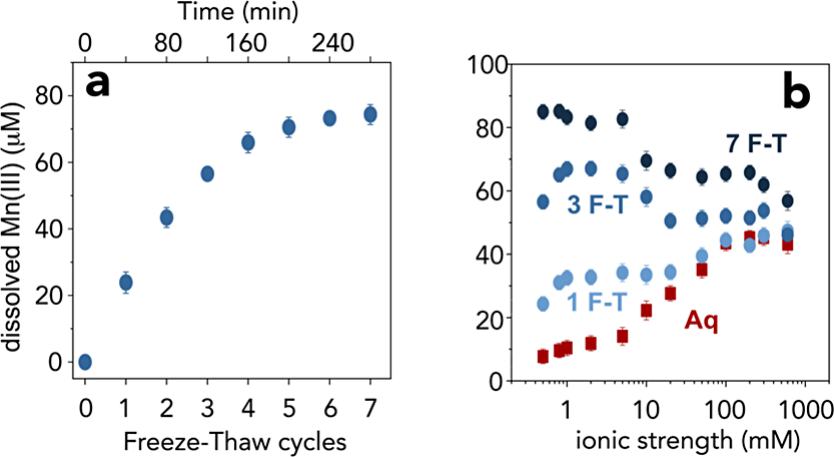
Mn(III) formation by freeze–thaw (F–T) cycling of pH_ini_ = 7 ± 0.05 suspensions of 50 μM MnO_2_ (4.35 mg/L), 50 μM Mn^2+^ and 250 μM PP in (a) 0.5 mM NaCl and (b) 0.5–500 mM NaCl. Each 40 min cycle comprised a 20 min period of reaction in liquid water (25 °C) followed by a 20 min period in frozen water (−20 °C).

To test this idea of reagent trapping by ice, we tracked Mn(III) formation through a sequence of 7 freeze–thaw (F–T) cycles. These experiments (Figure 3a) revealed that each freeze event produced progressively more Mn(III) until the seventh F–T cycle, where concentrations plateaued at ∼80 μM Mn(III). This amounts to ∼8 times more Mn(III) than in liquid water at 25 °C (∼10 μM, Figure 1a), and to ∼80% of the Mn in the system. These systematic hikes in Mn(III) concentration align with those seen in the ∼40 min of freezing in the time-resolved experiments (Figure 1a). They can thus be most readily explained by reaction of species that were previously trapped in ice, and subsequently released during thawing events. This interpretation aligns with one of the very few other published studies on heterogeneous cryo-redox reactions,^71^ which showed that F–T cycling promoted the oxidative transformation of iodine by Fe(III) into reactive iodine, and of this iodine converted to organoiodine when reacted with natural organic matter.

To explore the salt-dependence on the freezing-driven formation of Mn(III), we performed an additional set of F–T experiments covering the 0.5–500 mM NaCl range (Figure 3b). We focused on results after the 1^st^, 3^rd^ and 7^th^ F–T cycles (Figure 3a). These experiments revealed that freezing enhanced Mn(III) formation at up to ∼50 mM NaCl after 1 F–T cycle, and at all NaCl concentrations after 3 and 7 F–T cycles. Additionally, while Mn(III) yields scaled with NaCl content after 1 F–T cycle, those obtained after 3 and 7 F–T cycles were smaller the higher the NaCl content. Still they were consistently higher than in liquid water.

We explain these results by the competing effects of (i) chloride-promoted redox reactions,^63−65^ and (ii) the salt-dependent availability of liquid water in the LIB. Chloride-promoted reactions, which can clearly be appreciated in liquid water, were best manifested in ice after 1 F–T cycle. Here, freezing promoted Mn(III) formation by increasing the chloride content in the LIB, where the reactions took place. This freeze concentration effect was most effective in dilute NaCl because relative changes in LIB NaCl concentrations were greatest.^51^ As a result, this effect became attenuated at high salt content, chiefly explaining the identical yields after 1 F–T cycle and those achieved in liquid water. Compounded on this effect, is the loss of LIB liquid water by the formation of the cryosalt mineral hydrohalite (NaCl·2H_2_O), whose growth withdraws liquid water from the LIB.^72^ We can support this claim using Raman microscopy (Figure S7) showing that hydrohalite formed in the LIB at the higher salt content. This salt-driven removal of water from the LIB can thus explains the smaller Mn(III) yields at this ionic strength, and as especially observed after 3 and 7 F–T cycles.

**Figure 4:**
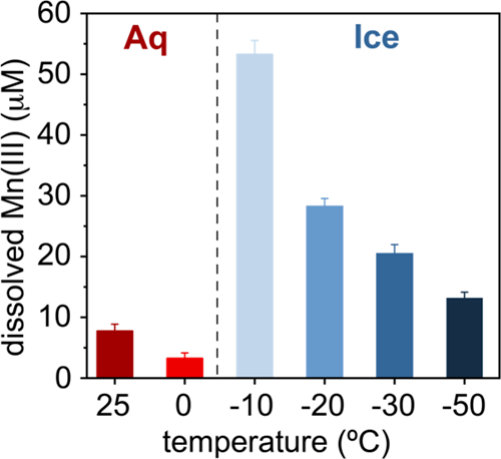
Temperature effects on dissolved Mn(III) production in liquid water (Aq) and ice (Ice). Mn(III) produced in 2 h of reaction by comproportionation of 50 μM MnO_2_ (4.35 mg/L) and 50 μM Mn^2+^ in the presence of 250 μM PP in solutions of 0.5 mM NaCl at pH_ini_ = 7 ± 0.05.

In an effort to test how temperature and availability impact Mn(III) formation, we compared Mn(III) yields in warm (25 °C) and cold (∼0 °C) liquid water, then in ice at the temperatures crossing the eutectic (−21.3 °C), down to −50 °C (Figure 4). These experiments confirmed again the strong enhancement of Mn(III) generated in ice in relation to liquid water. The strongest yields were at −10 °C, where liquid water coexisted with ice.^73^ Again, we attribute this enhancement to the accumulation of reactive MnO_2_, Mn^2+^ and PP in the LIB. While this notion for a sustained activity of ice below the eutectic clashes with the classical idea that reactivity should have been suppressed,^51^ earlier^74,75^ and an increasingly growing body of evidence^76−78^ are lending support to the idea that small persistent populations of liquid water can be preserved in the form of (i) supercooled intergrain boundary water^73,79^ resulting from the freezing process, and (ii) quasi-liquid layers at surfaces of ice microcrystals and MnO_2_ particles.^80,81^ These are certainly avenues worthy of investigation in forthcoming studies, and which can lead to new ideas of geochemical reactions at extremely low temperatures.

## 4 Environmental Implications

We showed that freezing greatly accelerated the formation of dissolved Mn(III)–PP complexes from the comproportionation between solid MnO_2_ and dissolved Mn(II) species. Freeze concentration of reactants in the LIB of ice was proposed to be the main driving force for Mn(III) formation. Seven consecutive freeze–thaw cycles led to the conversion of up to ∼80% of all Mn into Mn(III) species at low ionic strengths. Conversion at high ionic strength was, in contrast, not at high but promoted by Cl complexation.^63−65^

This study on the impact of freezing on dissolved Mn(III) formation revealed that freezing can be an important formation pathway contributing to natural pools of soluble Mn(III) in nature. Although this work focused on comproportionation reactions in the presence of only pyrophosphate, our findings can be generalized to other Mn(III)-stabilizing (in)organic ligands. In particular, these can include natural organic matter, which possesses Mn(III)-complexing chelating functional groups.

Our findings may consequently imply that soluble Mn(III) aquatic and estuary settings in middle- to high-latitude regions could be catalyzed by freeze–thaw episodes. This idea could potentially help explain recently reported high fluxes of dissolved Mn in creeks and estuaries during Spring snowmelt events.^82^ It could also help understand the coupled biogeochemical cycling of Mn with those of carbon, nitrogen, sulfur, oxygen, and iron.^33,44,45^ Recognition of the impact of freezing on the biogeochemical cycling of Mn may even shed new light on these cycles, especially that the dynamics of the hydrosphere and cryosphere are increasingly altered by climate change.

## Supporting Information Available

Time profile of Mn(III) formation in ligand-assisted dissolution of MnO_2_ or abiotic oxidation of Mn^2+^; ionic strength-dependence on zero-order rate constants; aqueous speciation of Mn^2+^ and PP over pH; distribution of dissolved Mn species after reactions in liquid water and ice; XPS spectra of reference Mn-phases; Raman microscopy image and spectra of ice microcrystals and the LIB; kinetic model parameters; and thermodynamic constants for the aqueous Mn and PP species (PDF)
