# Supplementary material for: Redox-Driven Formation of Mn(III) in Ice
Source: Environ Sci Technol. 2024 Aug 17;58(34):15194–201. doi: 10.1021/acs.est.4c03850 (PMC11360366; doi:10.1021/acs.est.4c03850)
Supplement: Supplementary file 1 — es4c03850_si_001.pdf [file es4c03850_si_001.pdf]

SUPPLEMENTARY MATERIALS

Redox-driven formation of Mn(III) in ice

Tao Chen[1], Tra My Bui Thi[1], Tao Luo[1], Wei Cheng[2], Khalil Hanna[1], Jean-François Boily[3,*]

[1] Univ. Rennes, École Nationale Supérieure de Chimie de Rennes, CNRS, ISCR-UMR 6226, F-35000 Rennes, France

2 College of Resources and Environmental Science, South-Central University for Nationalities, Wuhan 430074, P. R. China

[3] Department of Chemistry, Umeå University, SE-901 87, Umeå, Sweden

**corresponding author: jean-francois.boily@umu.se*

Summary: 13 Pages, 7 figures, 3 tables.

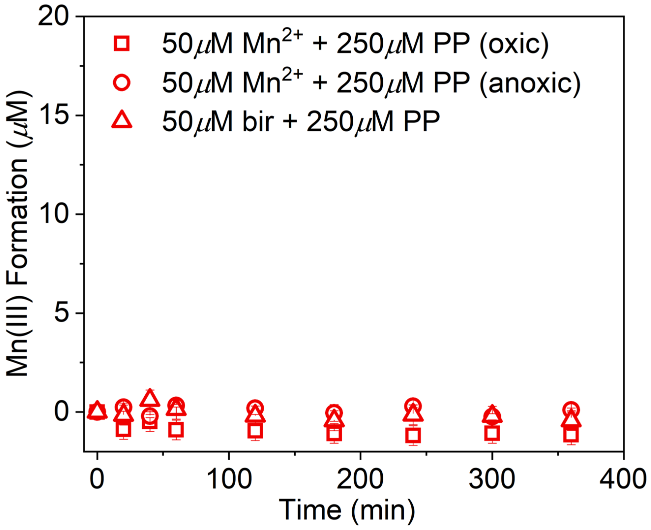

Figure S1. Time-resolved formation of Mn(III) in ligand-assisted dissolution of MnO_2 or abiotic oxidation of Mn^{2+} under oxic and anoxic conditions at pH 7 ± 0.05 in aqueous solutions at 25°C.

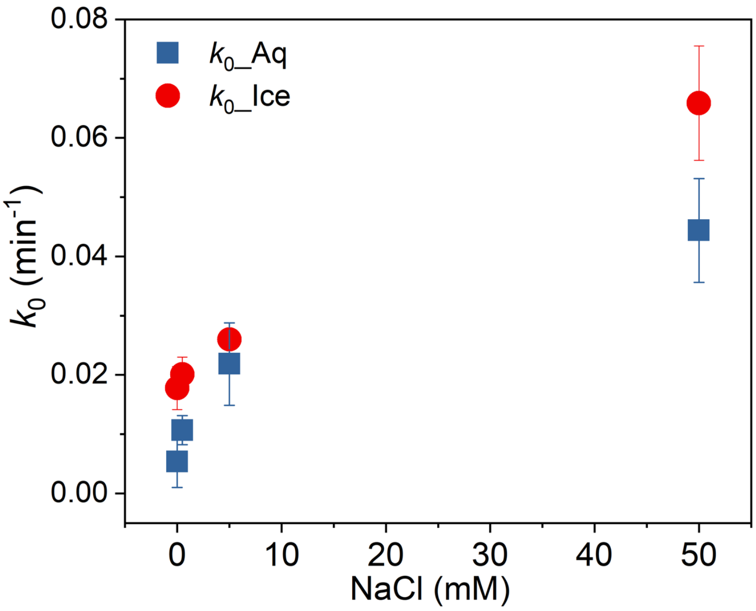

Figure S2. Variations in zero-order rate constants (k_0) of Mn(III) formation by comproportionation of 50 μM MnO_2 (4.35 mg/L) and 50 μM Mn^{2+} in the presence of 250 μM PP at pH_{ini} = 7 ± 0.05 in liquid water (Aq) or frozen (Ice) water.

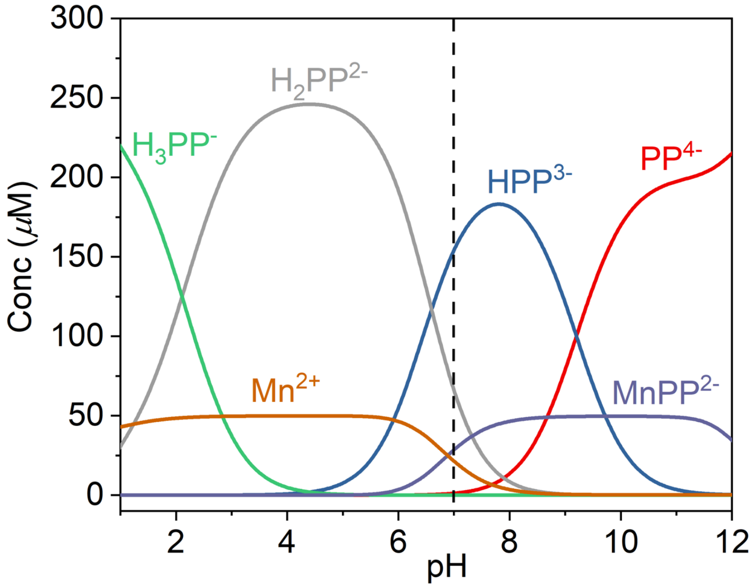

Figure S3. Aqueous speciation equilibrium of 50 μM Mn^{2+} and 250 μM PP as a function of pH.

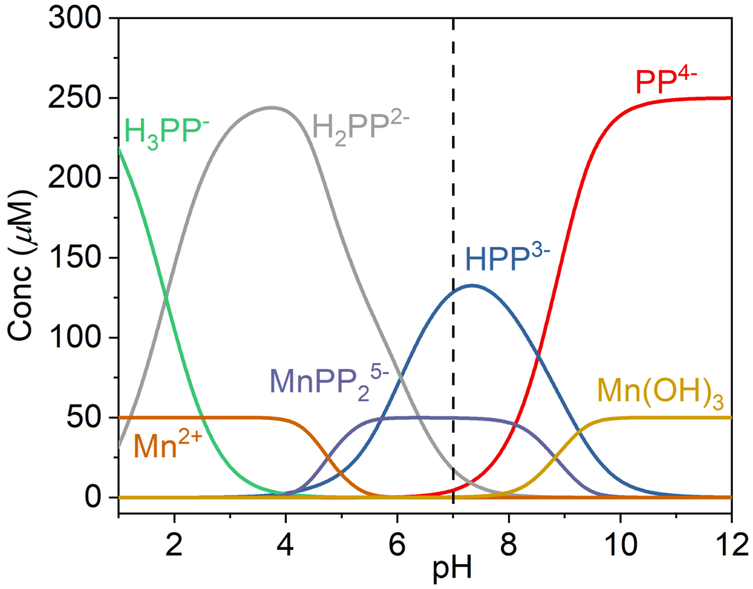

Figure S4. Aqueous speciation equilibrium of 50 μM Mn^{3+} and 250 μM PP as a function of pH.

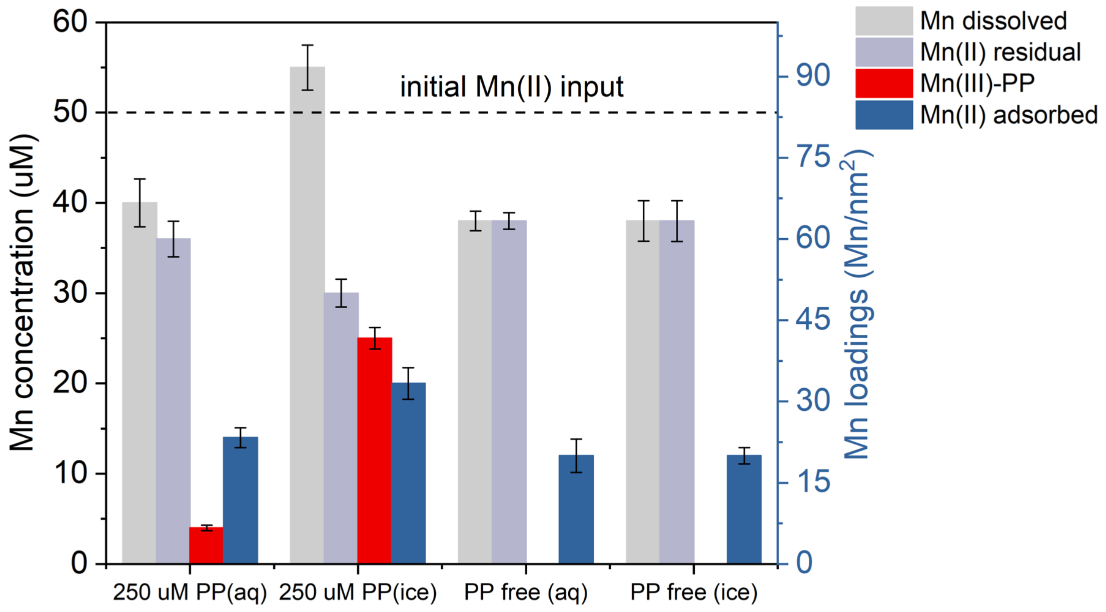

Figure S5. Distribution of dissolved Mn species and Mn loadings after Mn(II) (50 μM) in equilibrium with MnO_2 (50 μM) in the presence or absence of PP (250 μM) in liquid water (Aq) at 25°C and in ice (Ice) at -20 °C at pH_{ini} = 7 ± 0.05 after 1 h of reaction.

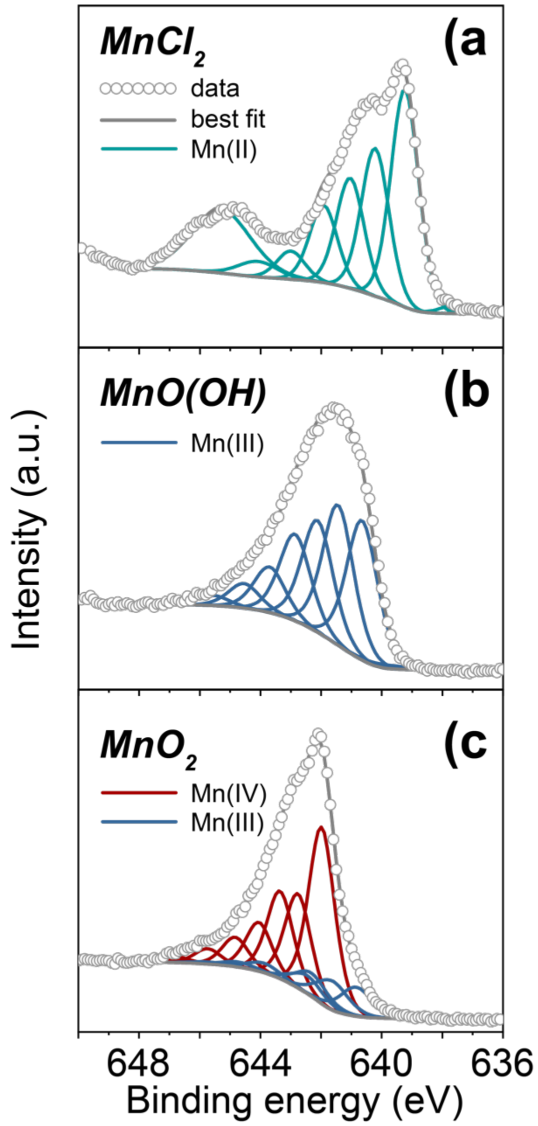

Figure S6. XPS spectra and fit results of Mn2$p_{3/2}$ region of the standards: (a) $MnCl_2$, (b) Manganite(MnOOH), and (c) pyrolusite (MnO_2).

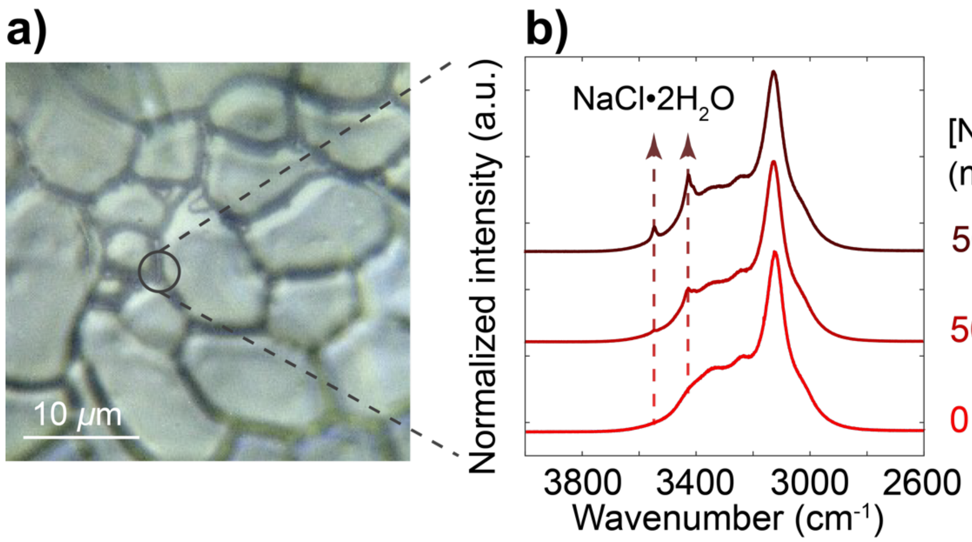

Figure S7. (a) Optical image of a frozen 50 mM NaCl solution at -20°C. (b) Raman spectra in the OH stretching region of frozen saline waters of various salinity at -20°C. The intensity of each spectrum is normalized to the 3130 cm^{-1} peak. The growth of cryosalt hydrohalite ($NaCl \cdot 2H_2O$) as a function of NaCl concentration is indicated by arrows.

Table S1. Fitting parameters of kinetics model for soluble Mn(III) formation in frozen and aqueous solutions.

IS (mM)	$[\mathrm{Mn(III)-PP}]_\infty$ (μM)	k_1 (min^{-1})	k_0 (min^{-1})	R^2
	Ice			
0	21.3 ± 0.83	0.108 ±0.019	0.018 ± 0.004	0.98
0.5	22.6 ± 0.67	0.109 ±0.015	0.020 ± 0.003	0.99
5	25.2 ± 0.41	0.082 ±0.005	0.026 ± 0.002	0.99
50	33.4 ± 0.80	0.042 ±0.005	0.066 ± 0.010	0.99
	Aq			
0	9.3 ± 1.84	0.008 ±0.002	0.005 ± 0.004	0.99
0.5	9.7 ± 0.99	0.009 ±0.001	0.011 ± 0.002	0.99
5	12.7 ± 1.98	0.022 ±0.006	0.022 ± 0.007	0.98
50	29.6 ± 0.43	0.033 ±0.002	0.044 ± 0.009	0.99

Table S2. Equilibrium reaction and constants related to the Mn thermodynamic calculations.

Species	Equilibrium reactions	Log *K*	Ref
PP^{4-}	$PP^{4-} = PP^{4-}$	0	-
HPP^{3-}	$PP^{4-} + H^+ = HPP^{3-}$	9.40	1–3
H_2PP^{2-}	$PP^{4-} + 2H^+ = H_2PP^{2-}$	6.70	1–3
H_3PP^-	$PP^{4-} + 3H^+ = H_3PP^-$	2.28	1–3
Mn^{2+}	$Mn^{2+} = Mn^{2+}$	0	-
$Mn(OH)^+$	$Mn^{2+} + H_2O = Mn(OH)^+ + H^+$	-10.59	4
$Mn(OH)_{2(aq)}$	$Mn^{2+} + 2H_2O = Mn(OH)_2 + 2H^+$	-22.20	4
$Mn(OH)_3^-$	$Mn^{2+} + 3H_2O = Mn(OH)_3^- + 3H^+$	-34.80	4
$Mn(OH)_4^{2-}$	$Mn^{2+} + 4H_2O = Mn(OH)_4^{2-} + 4H^+$	-48.30	4
$MnPP^{2-}$	$Mn^{2+} + PP^{4-} = MnPP^{2-}$	6.51	5
Mn^{3+}	$Mn^{2+} = Mn^{3+} + e^-$	-25.51	1–3
$MnOH^{2+}$	$Mn^{3+} + H_2O = MnOH^{2+} + H^+$	0.27	1–3
$Mn(OH)_{3(c)}$	$Mn^{3+} + 3H_2O = Mn(OH)_3 + 3H^+$	-6.3465	1–3
$MnPP^-$	$Mn^{3+} + PP^{4-} = MnPP^-$	16.7	6
$MnPP_2^{5-}$	$Mn^{3+} + 2PP^{4-} = MnPP_2^{5-}$	28.9	7
MnH_2PP^+	$Mn^{3+} + PP^{4-} + 2H^+ = MnH_2PP^+$	21.1	6
$MnH_4PP_2^-$	$Mn^{3+} + 2PP^{4-} + 4H^+ = MnH_4PP_2^-$	40.4	6
$MnH_6PP_3^{3-}$	$Mn^{3+} + 3PP^{4-} + 6H^+ = MnH_6PP_3^{3-}$	59.2	6

Table S3. Fit parameters from $Mn2p_{3/2}$ spectra of the standards.

	P1	P2	P3	P4	P5	P6	P7	P8
$MnCl_2$-Mn(II)								
BE (eV)	639.24	640.22	641.04	641.92	643	644.09	645.29	637.59
FWHM (eV)	1.08	1.08	1.08	1.08	1.08	1.51	2.29	1.08
I(re)	1.00	0.680	0.507	0.355	0.130	0.100	0.615	0.039
G/L	30	30	30	30	30	30	30	30
Manganite-Mn(III)								
BE (eV)	641.44	640.65	642.12	642.86	643.7	644.54	645.56	639.85
FWHM (eV)	1.21	1.21	1.21	1.21	1.21	1.21	1.21	1.75
I (re)	1.00	0.995	0.798	0.618	0.326	0.175	0.073	0.036
G/L	50	50	50	50	50	50	50	50
Pyrolusite-Mn(IV)								
BE (eV)	641.98	642.76	643.35	644.05	644.83	645.74	646.83	647.05
FWHM (eV)	1.02	1.02	1.02	1.02	1.02	1.02	1.02	1.02
I (re)	1.00	0.534	0.496	0.277	0.167	0.087	0.036	0.013
G/L	50	50	50	50	50	50	50	50

References

(1) Morel, F. M. M.; Hering, J. G. *Principles and Applications of Aquatic Chemistry*; John Wiley & Sons, 1993.

(2) Smith, R. M.; Martell, A. E.; Motekaitis, R. J. NIST Standard Reference Database 46. *NIST Crit. Sel. Stab. constants Met. complexes database Ver* **2004**, *2*.

(3) Gustafsson, J. P. Visual MINTEQ, Ver. 3.1, Department of Land and Water Resources Engineering, Royal Institute of Technology-KTH, Stockholm, Sweden.

(4) Morgan, J. J. Kinetics of Reaction between O_2 and Mn(II) Species in Aqueous Solutions. *Geochim. Cosmochim. Acta* **2005**, *69* (1), 35–48.

(5) Bilinski, H. Precipitation and Complex Formation in the System $Mn(ClO_4)_2$-$Na_4P_2O_7^-$-pH (295 K, *I*= 0.5 and *I*≈ 0 Mol Dm^{-3}). *Polyhedron* **1983**, *2* (5), 353–358.

(6) Gordienko, V. I.; Sidorenko, V. I.; Mikhailyuk, Y. I. Amperometric Investigation of Mn (III) Pyrophosphate Complexes. *Russ J Inorg Chem* **1970**, *15*, 1241–1244.

(7) Qian, A.; Zhang, W.; Shi, C.; Pan, C.; Giammar, D. E.; Yuan, S.; Zhang, H.; Wang, Z. Geochemical Stability of Dissolved Mn(III) in the Presence of Pyrophosphate as a Model Ligand: Complexation and Disproportionation. *Environ. Sci. Technol.* **2019**, *53* (10), 5768–5777.
